# Perceptions of improvements and mental health outcomes of micro-renewal in old Danwei community: a survey of residents in Hengyang, China

**DOI:** 10.3389/fpubh.2024.1419267

**Published:** 2024-11-01

**Authors:** Mengjia Chen, Lei Shi, Bo Wang, Hao Sun, Dahu Lin, Yating Chang, Shuting Yan, You Peng, Tao Feng

**Affiliations:** ^1^The School of Architecture and Art, Central South University, Changsha, China; ^2^School of Architecture, South China University of Technology, Guangzhou, China; ^3^Urban Planning and Transportation, Department of the Built Environment, Eindhoven University of Technology, Eindhoven, Netherlands; ^4^Urban and Data Science Lab, Graduate School of Advanced Science and Engineering, Hiroshima University, Higashi Hiroshima, Japan

**Keywords:** mental health, perception of environmental improvements, micro-renewal, entropy-TOSIS, multinominal logit

## Abstract

**Introduction:**

Danwei communities are a testament to China’s socialist urban development, characterized by the self-sufficiency, strong social networks, and institutional management. In the historical context of urban development in China, many old communities have evolved from former housing areas of Danwei. After more than 40 years of use, the buildings, and environments in the old Danwei communities remain in disrepair, dirty, disorderly and poor condition. Many functions have failed that negatively affect the quality of life and health of residents. After Covid-19, improving the mental health of residents has become a major goal of public policies in various countries.

**Methods:**

To explore the residents’ mental health in the micro-renewal, this study carried out a survey regarding residents’ evaluation on the micro-renewal and their mental health in two renewed Danwei communities in Hengyang, China. More than 800 respondents joined the interview, among them, 634 samples are effective for analysis. Entropy-TOPSIS is applied to analyze the weights of various indicators of micro-renewal. And multinominal logit model is used to examine the relationship between the personal factors, satisfaction on various items of micro-renewal and mental health.

**Results and Discussion:**

The findings indicates that the mental health level of residents living in Danwei community is affected by micro-renewal. The socio-demographic characteristics and behavior factors can influence on the possibility of being in different levels of mental health. The satisfaction on the components of micro-renewal and improvements after micro-renewal is also determiner of residents’ mental health level. In addition, the heterogeneity is address in mental health.

## 1 Introduction

The rapid and unplanned urbanization is one of the main ecological and human challenges in the 21st century (1). The instability of the urban system has been intensified along with climate change and environmental deteriorating through the increasing anthropological activities. The World Health Organization (WHO) put forward the initiative aiming at improving the health and well-being of urban population through participatory and multi-sectoral urban governance by 2028, and proposed that health does not start from hospitals or clinics, but from our families and communities (2). To support the sustainability in economic and social development and improve the environmental quality and population’s health in urban areas, urban renewal has been carried out in various ways all over the world (3–8).

The urbanization process in China has been significantly accelerated in the last decades. The population living in cities was about 69.79 million in 1978, which accounted for 17.9% of the total population in China at that time. By the end of 2022, this proportion increased to 65.22%, in other words, more than 920 million population in China is living in cities now. However, in the wake of urban development and population growth, the shortcomings are becoming apparent in the existing large number of old communities (3, 9). These old communities were typically built by state-owned enterprises, government departments, and public institutions to house their employees and their families. The term “Danwei” refers to all these organizations that provided employment, housing and various social services to its members. Danwei has been the principal social organizational form in Chinese socio-economic contexts until the beginning of 21^st^ century (10–13).

The old Danwei communities in China, once the cornerstone of socialist urban development, are now facing significant challenges as they age. There is a serious quality conflict between old Danwei communities and new communities, which exacerbates social inequality in housing and hinders sustainable urban development (10). Compared with newly built communities, there are limitations such as incomplete supporting facilities, serious illegal constructions, and insufficient parking spaces. Moreover, after more than 40 years of use, the buildings in the old communities remain in disrepair, dirty, disorderly and poor condition. Many functions have failed that negatively affect the quality of life of residents and impact on their health. Such physical conditions of old Danwei communities can significantly affect residents’ mental well-being (11). Living in poorly maintained environments can lead to feelings of helplessness and despair, exacerbating stress and anxiety (12). The lack of investment in these communities also means that essential services, such as sanitation and waste management, are often subpar, further degrading the living conditions and contributing to the residents’ mental strain. The residents, especially the older adults and children, find themselves confined to environments that do not support their developmental and social needs, leading to increased incidences of depression (13). Perhaps one of the most insidious issues facing residents of old Danwei communities is social isolation. As these communities were originally built for the employees of specific factories or institutions, the social fabric was once tightly knit. However, with the closure of many of these workplaces and the outmigration of younger populations, the remaining residents often find themselves isolated. The breakdown of these social networks means that many residents, particularly the older adults, experience loneliness, which has been strongly linked to poor mental health outcomes (14–16).

In recent years, to achieve fair health and social outcomes, the renewal of old residential community has become the key part of urban renewal where the obvious population aggregation, regionality and symbiosis exist (10, 17, 18). Improving the mental health of residents has become a major goal of public policies in various countries, especially in urban and rural development policies. The local government, scholars and urban planning and public health experts have been exploring ways to improve residents’ mental health through the intervention of old residential community renewal (8, 19–22). Based on literature regarding the research on urban renewal, the large-scale demolition and renovation are not suitable model for resilience of old Danwei communities.

An innovative renovation measure was firstly proposed as “Micro-renewal” by urban renewal bureau of Guangzhou in 2016. Compared with the large-scale and government led renovation model, it is a people-oriented urban renewal model with minor rebuild (23). Compared to reconstruction, micro renovation is less likely to disrupt residents’ neighborhood attachment (24). At the same time, micro renovations contribute to the sustainable protection of living heritage, helping old Danwei communities retain their historical and collective memory related features and values while seeking development and adaptation (25). The micro-renewal, as an innovative approach, has been implemented in urban redevelopment (26, 27). It is the adjustment and functional replacement of the environment and suitable for places with inconsistent usage and poor environmental conditions (26).

The participation in planning and design of community renewal can affect residents’ sense of empowerment, thereby achieving higher health benefits (Baba, Kearns, McIntosh, Tannahill and Lewsey) (28). The focus of public participation is to safeguard the interests and demands of the public as well as the public interests of the community. Therefore, the impacts of public participation in micro-renewal of old Danwei communities on the mental health of residents are reflected in two aspects. On the one hand, micro-renewal can change the quality and appearance of houses and community, potentially affecting the emotions of residents; on the other hand, residents can promote the goal of amending the environment of community closer to their needs through public participation, while also improving their sense of belonging and cohesion toward the community, thereby enhancing their mental health and sense of happiness in life.

To explore the residents’ mental health in the renovation of old residential areas, self-assessment of their expected mental health is necessary (29). However, current research on urban renovation and resilience mainly focuses on quantifying the improvement of the physical environment and the psychological satisfaction of residents after renovation. Limited empirical evidence on the improvement of residents’ psychological health through urban renewal, especially microscale renovation, was presented (6, 20). It is necessary to extend the residents’ assessments on the improvements in micro-renewal with a reasonable self-assessment of the mental health for fully understanding the effects of micro-renewal of neighborhood. To achieve this goal, this study, therefore, takes two old Danwei community micro-renewal projects as cases to investigate the potential impacts of neighborhood renovation on the mental health of residents. The key variables in that affect the mental health of residents was identified to construct a comprehensive framework for post occupancy evaluation in neighborhood micro-renewal. The findings are expected to guide the sustainable development of old Danwei community in contexts of Chinese cities.

This study is aiming to examine the nature and strength of the relationships between residents’ satisfaction on neighborhood scale renewal and their mental health. The conceptual framework of post occupancy evaluation advocates that the transformation of the architectural environment will have an impact on the actual living experience of residents in various aspects in terms of environment, architecture, and resources, as well as personal psychology. Accordingly, the implementation of old Danwei community micro-renewal may affect the psychological health of residents through changes in different dimensions such as residential conditions, community environment, community safety, esthetics, and quality of activity venues, etc. The satisfaction evaluation which measures the benefits generated by micro-renewal can be obtained from the field surveys on residents. Therefore, this study proposes an innovative framework of “public participation-micro-renewal of old community-residents’ mental health.” The analysis framework links objective living conditions with mental health through micro-renewal of old Danwei communities and makes public participation an important prerequisite for the implementation of micro renovation projects.

## 2 Literature review

Urban renewal has been a significant focus of urban planning and public health research for decades, driven by the need to address the challenges posed by aging urban infrastructure, particularly in rapidly urbanizing countries like China. The old Danwei communities have become increasingly problematic, which are characterized by deteriorating infrastructure, outdated facilities, and an aging population, which together contribute to a range of social and health-related issues.

Previous studies have established a clear link between the quality of the built environment and mental health outcomes. Evans discusses how poor housing conditions, including inadequate lighting, poor ventilation, and structural decay, can exacerbate stress and anxiety among residents (30). Galea et al. further emphasize that living in deteriorating environments can lead to increased incidences of depression, particularly in low-income populations that lack the resources to move to better housing (13).

The inadequacy of facilities in aging communities has also been well documented. Li and Wu highlight that in many of China’s older urban neighborhoods, including Danwei communities, the lack of modern amenities such as healthcare services, recreational spaces, and social services severely impacts residents’ quality of life (15). This deficiency is particularly acute for the older adults, who are more dependent on local services for their daily needs. The absence of adequate facilities not only limits physical health but also contributes to social isolation, further exacerbating mental health issues.

Social isolation has been identified as a critical factor influencing mental health, particularly in older populations. Cacioppo and Cacioppo discuss the “toxic effects” of perceived social isolation, which can lead to increased risks of depression and anxiety (16). In the context of Danwei communities, where the social fabric has been eroded due to the outmigration of younger populations and the closure of many original workplaces, the remaining residents often find themselves isolated. This social isolation is compounded by the physical deterioration of the environment, creating a feedback loop that further degrades residents’ mental health.

Recent studies on urban renewal have begun to explore the potential benefits of interventions such as micro-renewal in improving both the physical environment and the mental health of residents (17, 31). Micro-renewal, as an innovative approach, focuses on small-scale, community-driven improvements that can enhance living conditions without the disruption associated with large-scale redevelopment. Zhao et al. provide a comprehensive analysis of micro-renewal projects across China, demonstrating their potential to improve residents’ quality of life by addressing both physical and social dimensions of urban living (23). Sheng et al. clarified the relationship between the development of urban community cultural heritage and the consolidation of local residents’ cultural identity (32). Tian et al. explored the role of participatory electronic planning models in old residential area renewal projects (33). Considering that the ultimate goal of renovating old residential areas is to improve the living conditions, health status, and well-being of residents, and achieve sustainable development, evaluating the impact of micro renovation projects on residents is the focus of research (31, 34). Liu and Li established 32-item index for five aspects, including politics, economy, culture, society, and ecology (26). Wang et al. established a 16-element index in terms of the benefits of government, resident, and developer, and constructed a comprehensive evaluation model for decision-making on urban renewal mode selection (35). Riera Pérez et al. developed a spatial decision support system for neighborhood-scale renewal projects which takes the actual situation of old community and the expected long-term resilience into account (36).

While there is substantial literature on the general impacts of urban renewal, there is limited empirical research specifically focusing on the mental health outcomes of micro-renewal in old Danwei communities. Most existing studies either address the physical improvements brought by urban renewal or discuss mental health in broader terms, without linking these two aspects in a comprehensive framework. This study aims to fill this gap by investigating how micro-renewal in old Danwei communities affects residents’ mental health. The research question guiding this study is: “How does micro-renewal impact the mental health of residents in old Danwei communities, and what are the key factors that mediate this relationship?”

By addressing this research question, the study aims to contribute to the growing body of knowledge on urban renewal and public health, offering insights that can inform future policy and practice in the renewal of aging urban communities.

## 3 Methodology

### 3.1 Data collection

**Figure 1:**
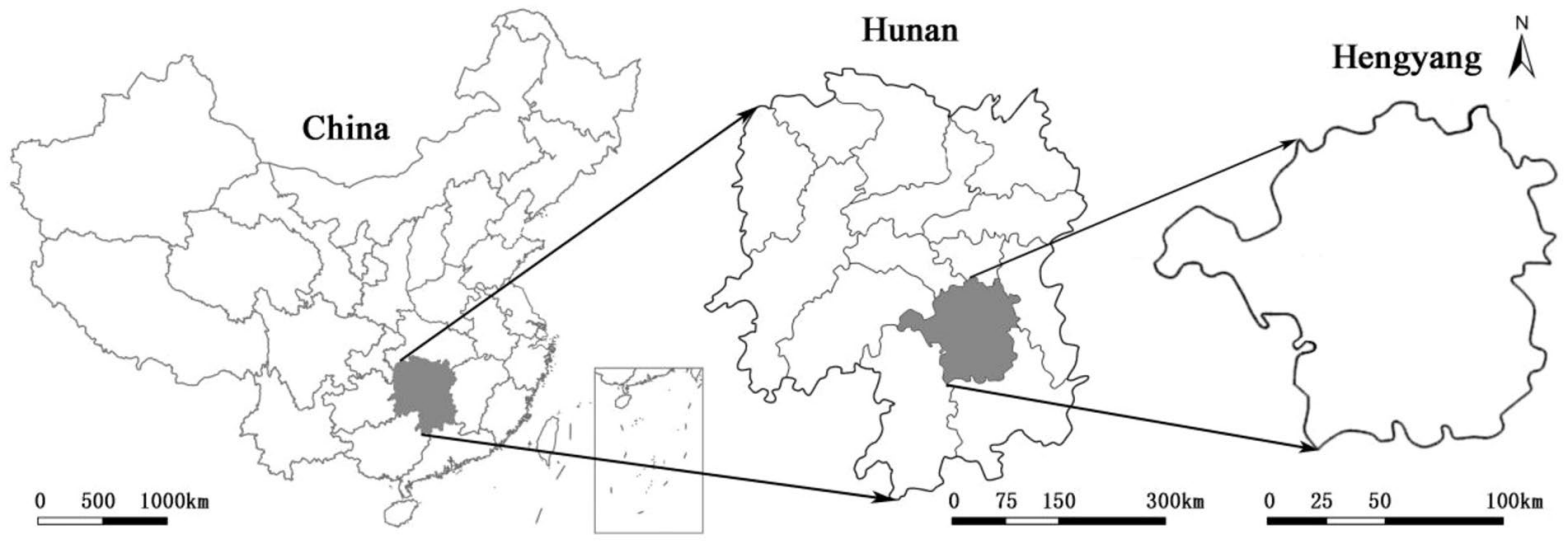
Location of Hengyang City.

The data collection was conducted in Hengyang, Hunan Province, China. By the end of 2022, there were more than 6.5 million permanent population living in Hengyang, which is the second largest city in Hunan Province. Hengyang is one of the important industrial cities in the central south of China (see Figure 1). Since the reform and opening up in China, many manufactures have been established in Hengyang. The thriving industry in the 1970s and 1980s had attracted a large population and built massive Danwei residential communities. At present, the communities left over from the planned economy era become outdated, incomplete functional facilities, low *per capita* land area, and poor environmental quality. However, these old Danwei communities preserve the collective memories of different generations and contain unique cultural values, making them unsuitable for major demolition and renovation. Therefore, it is urgent to apply micro-renewal to update them. The typical cases of renovations in Hengyang are suitable for studies on the mental health of residents after micro-renewal. In August 2023, a one-on-one questionnaire survey was conducted among all residents of two old Danwei communities in Hengyang, namely, Baizhuzao and Xinkuangcun. Baizhuzao community is the residential area of the former state-owned enterprise, which is classified as Community 1 (C1), and. Xinkuangcun community is the residential areas of a cable factory and a plastic factory which is classified as Community 2 (C2).

Baizhuzao is a residential area originally built by a former state-owned enterprise. It represents a typical old Danwei community where the majority of the infrastructure has significantly aged, and the social structure is relatively homogeneous. The community’s residents largely consist of retired workers who have lived in the area for decades, making it an ideal case to study the impacts of micro-renewal on a population that is deeply attached to its living environment and highly affected by changes in it. Xinkuangcun, on the other hand, is a residential area associated with a cable factory and a plastic factory, showcasing a different industrial background and a somewhat more diverse social structure. This community, while also an old Danwei community, has experienced different patterns of urban development and resident demographics compared to Baizhuzao. This diversity allows for a comparative analysis of how micro-renewal impacts communities with varying social dynamics and histories.

The questionnaire consists of questions regarding respondents’ personal basic information, housing conditions, psychological health assessment, and satisfaction on micro-renewal of the old Danwei communities. The Short Warwick Edinburgh Mental Well-being Scale (SWEMWBS) was used for examining the respondents’ mental health (34). SWEMWBS has been extensively validated and can be used in various regions, languages, and cultural backgrounds, as well as in various built environment projects. The scale has a total of 14 items, with a rating range of 1–5 points for each item. The total score for each item is calculated by adding up the total score (ranging from 14 to 70 points). This study used K-means clustering to input the scores of each item in each sample, and ultimately divided the scores into high, average, and low mental health. Among them, the score range for groups with low mental health levels is “32–51″, the score range for groups with average mental health levels is “49–60″, and the score range for groups with high mental health levels is “60–70″. Respondents’ subjective evaluation was estimated by the Likert 7-point scale.

**Figure 2:**
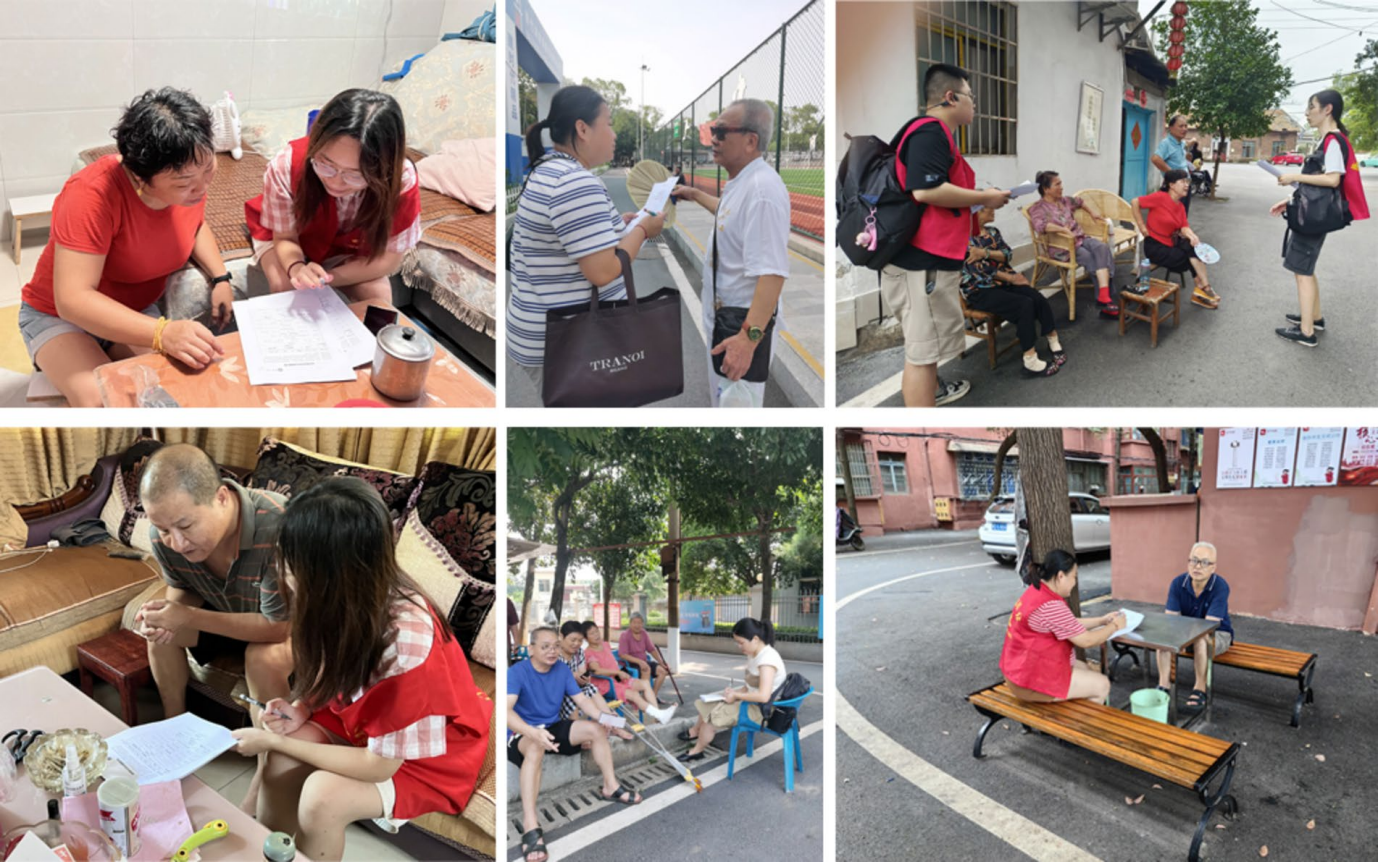
The scenes of interviews in the two old Danwei communities.

Before the field investigation, seven research assistants were trained to ensure consistency and accuracy in data collection, and how to administer the questionnaire, handle respondents’ queries, and record responses accurately. A random sampling method was used to select participants. The sampling frame included all households in the two old Danwei communities, where improvements were made to residential buildings and urban surroundings, ensuring that every resident had a known and non-zero chance of being selected. During the survey, we also got some help from the neighborhood management commission to invite residents to join in the interview. The scenes of interviews are shown in Figure 2.

The structured interview was carried out at convenient locations for residents, such as community centers, public spaces, or respondents’ homes if they preferred. Each interview takes approximately 20 min. Interviews were conducted face-to-face, with trained interviewers asking standardized questions to evaluate residents’ perceptions and mental health outcomes. During the survey, the interviewer explained the questionnaire to the interviewee to ensure they could understand and answer accurately. The total number of households in two communities is 1,261 (C1:936, C2:325). After a two-week (from 10th to 23rd August 2023) on-site survey, a total of 634 (503 in C1 and 131 in C2) effective questionnaires were collected.

### 3.2 Entropy-TOPSIS

The effectiveness of micro-renewal is mainly manifested in improving living conditions, optimizing outdoor environments, enhancing public services, and optimizing infrastructure. To analyze its effect on mental health of residents in depth, this study constructs an evaluation model with indicators regarding satisfaction on six key renewed components, including night lighting, facility, public space, safety, style and service. In this model, each component is characterized with specific measured items, which are shown in Table 1. In addition, the level of satisfaction of residents toward the improvements of micro-renewal is listed in Table 2.

**Table 1:** Satisfaction on the components after micro-renewal.

Target layer	Criterion layer	Indicator layer
Satisfaction level	Night lighting	Corridor lighting Street lighting
	Facilities	Staircase railing Roof waterproofing and drainage Road drainage and sewage
	Public space	Fitness venues and facilities Public activity spaces Parking spaces and facilities Greenery
	Security	Gate and access control Monitoring and security Firefighting equipment
	Appearance	External wall painting Residential corridor wall painting, Road pavement
	Service	Road system Garbage collection

**Table 2:** Satisfaction on the improvements after micro-renewal.

Target layer	Indicator layer
Satisfaction level	Improvement in sound environment
	Improvement in thermal environment
	Improvement in air quality
	Improvement in nighttime lighting
	Improvement in daily grocery shopping
	Improvement in services
	Improvement in public spaces
	Improvement in safety status

The method of modeling applied in this study combines Technique for Order Preference by Similarity to Ideal Solution (TOPSIS) and Entropy weight, namely Entropy-TOPSIS. As a multicriteria decision making method, the basic concept of TOPSIS is that the chosen alternative should have the shortest distance from the ideal solution and the farthest distance from the negative ideal solution. The Entropy-TOPSIS uses entropy value to determine the weights of various indicators and calculates the optimal and worst solutions of each evaluation object through TOPSIS. The concept of entropy originates from thermodynamics and is used to quantify the degree of chaos in a system. It can better reflect the interrelationships between the dimensions of comprehensive satisfaction and agreement. In this study, the weight of indicator is determined by the amount of information it provided. The larger the amount of information, the greater the utility of the indicator in the decision-making process. This method lies in determining the positive ideal solution (D+) and the negative ideal solution (D-), and then calculating the geometric distance between each alternative solution and these ideal solutions, characterizing the relative closeness of the evaluation object to the optimal solution, providing an intuitive and quantitative indicator for evaluating the effectiveness of micro-renewal in old Danwei communities. The steps are presented as follows:

Step 1: To establish a decision matrix.

An evaluation matrix ${\left(x_{ij}\right)}_{m\times n}$ consisting of m samples and n evaluation indicators.


(1) $$H={\left(x_{ij}\right)}_{m\times n},\left(i=1,2,\cdots ,m;\;j=1,2,\cdots ,n\right)$$

(2) $$X=\left[\begin{array}{ccc} x_{11} & \cdots & x_{1n}\\ \vdots & \ddots & \vdots \\ x_{m1} & \cdots & x_{mn} \end{array}\right]$$

where, *i* is the sample size, and *j* is the number of indicators. In Equations 1 and 2 of this study, m =634, and n =42, including 17 indicators of pre renewal conditions, 17 indicators of the post occupancy evaluation and 8 indicators of evaluation on the improvements after micro-renewal compared to before renewal.

Step 2: To normalize the decision matrix.

The indicators in this study are all positive, so there is no need for forward normalization. The normalized matrix ${\left(z_{ij}\right)}_{m\times n}$ is shown in Equations 3 and 4:


(3) $$z_{ij}=\frac{x_{ij}}{\sqrt{\sum_{i=1}^{m}x_{ij}^{2}}}$$

(4) $$Z=\left[\begin{array}{ccc} z_{11} & \cdots & z_{1n}\\ \vdots & \ddots & \vdots \\ z_{m1} & \cdots & z_{mn} \end{array}\right]$$

Step 3: To calculate information entropy. The calculation formulas are shown in Equations 5–7.


(5) $$P_{ij}=\frac{z_{ij}}{\sum_{i=1}^{m}z_{ij}}$$

(6) $$e_{j}=-k\overset{m}{\underset{i=1}{\sum }}P_{ij}\ln\left(P_{ij}\right),\left(j=1,2,\cdots ,n\right)$$

(7) $$k=\frac{1}{\ln m}$$

where, $p_{ij}$ represents the proportion of the score of the i-th sample under the j th indicator to that indicator; $e_{j}$ represents the information entropy of the j th indicator.

Step 4: To calculate entropy weight. The calculation formula Equation 8 is presented as follow:


(8) $$w_{j}=\frac{1-e_{j}}{\sum_{j=1}^{m}\left(1-e_{j}\right)}$$

where, $w_{j}$ is the entropy weight of the j th indicator.

Step 5: To define the maximum and minimum values of evaluation indicators.

According to the normalization matrix Z, the optimal and worst solutions for each evaluation indicator, the optimal solution vector and the worst solution vector are, respectively, represented in Equations 9–12, which are shown as:


(9) $$Z^{+}=\left(Z_{1}^{+},Z_{2}^{+},\cdots ,Z_{n}^{+}\right)$$

(10) $$Z^{-}=\left(Z_{1}^{-},Z_{2}^{-},\cdots ,Z_{n}^{-}\right)$$

(11) $$Z_{j}^{+}=\max\left(Z_{1j},Z_{2j},\cdots ,Z_{ij}\right),\left(i=1,2,\cdots ,m;\;j=1,2\text{,}\right)$$

(12) $$Z_{j}^{-}=\min\left(Z_{1j},Z_{2j},\cdots ,Z_{ij}\right),\left(i=1,2,\cdots ,\;m;\;j=1,\;2\text{,}\right)$$

where, $Z_{j}^{+}$ is the maximum value of the j th evaluation indicator, and $Z_{j}^{-}$ is the minimum value of the j th indicator.

Step 6: To calculate the weighted geometric distance between the evaluation object and the maximum and minimum values. The calculation formulas are shown in Equations 13 and 14.


(13) $$D_{i}^{+}=\sqrt{\overset{n}{\underset{j=1}{\sum }}w_{j}{\left(Z_{j}^{+}-Z_{ij}\right)}^{2}}$$

(14) $$D_{i}^{-}=\sqrt{\overset{n}{\underset{j=1}{\sum }}w_{j}{\left(Z_{j}^{-}-Z_{ij}\right)}^{2}}$$

where, $Z_{ij}$ is the normalized value of the i th individual for the j th evaluation indicator, $Z_{j}^{+}$ is the maximum normalized value of all the j th evaluation indicators, $Z_{j}^{-}$ is the minimum normalized value of all the j th evaluation indicators, and $w_{j}$ is the entropy weight of the j th evaluation indicator.

Step 7: To calculate relative closeness (overall satisfaction). The calculation formula is shown in Equation 15.


(15) $$C_{i}=\frac{D_{i}^{-}}{D_{i}^{+}+D_{i}^{-}}$$

where, the range of Ci values is [0, 1], and the closer it is to 1, the closer the evaluation object is to the optimal level, indicating a higher overall satisfaction of the sample. On the contrary, the closer to 0, the closer the evaluation object is to the worst level, indicating a lower overall satisfaction of the sample.

### 3.3 Multinomial logit model

The multinomial logit model (MNL) has been widely applied in multiple disciplines, including transportation, medicine, architecture, urban and rural planning, etc. MNL was used in this study to explore the influencing factors of positive mental health assessment. The dependent variable classification is expressed in Equation 16:


(16) $$y_{i}=\{\begin{array}{c} 1，\mathrm{if}\;y_{i}^{*}\leq \mu_{1}\\ 2，\mathrm{if}\mu_{j-1}\leq y_{i}^{*}\leq \mu_{j}\\ \vdots \\ J，\mathrm{if}\;y_{i}^{*}\geq \mu_{J-1} \end{array}$$

where $\mu_{j}$ is the threshold point of $y_{i}^{*}$ . The link function is the logit transformation. In this study, J =3 since each result of SWEMWBS was the dependent variable (3-level: high, average, and low). The link function is the logit transformation (38), which is expressed as Equation 17.


(17) $$\mathrm{logi}t^{-1}\left(y_{i}^{*}\right)=\frac{1}{1+\exp\left(-y_{i}^{*}\right)}$$

The probability $P=\left(y_{i}=j\right)$ for the i th individual being in the j th category is given by Equation 18:


(18) $$P\left(y_{i}=j\right)=\frac{\exp\left(\beta_{j}X_{i}\right)}{1+\sum_{k=1}^{j-1}\exp\left(\beta_{k}X_{i}\right)}$$

where j ranges from 1 to j−1 (in this case j=3 ), $\beta_{j}$ are the coefficients for the j th outcome, and $X_{i}$ is the vector of independent variables for the i th individual. 16 independent variables are respondents’ socio-demographic information, housing condition, and satisfaction evaluations. By conducting Chi-square tests on the dependent and independent variables, we removed variables that were not correlated.

Hausman test is applied for examining the independence irrelevant alternatives (IIA) of MNL, a step-by-step description of how we conduct it is shown as follows:

1. Estimate the multinomial logit model using all the available alternatives and obtain the coefficient estimates ${\hat{\beta }}_{\mathrm{full}}$ and the variance–covariance matrix $Var\left({\hat{\beta }}_{\mathrm{full}}\right)$ .
2. Estimate the multinomial logit model by excluding one of the alternatives and obtain the coefficient ${\hat{\beta }}_{\mathrm{restricted}}$ and the variance–covariance $Var\left({\hat{\beta }}_{\mathrm{restricted}}\right)$ .
3. The test statistic is based on the difference between the estimated coefficients from the full model and the restricted model. The formula for the Hausman test statistic (39) is Equation 19.

(19) $$\begin{array}{l} H=\\ \left({\hat{\beta }}_{\mathrm{full}}-{\hat{\beta }}_{\mathrm{restricted}}\right){\left[Var\left({\hat{\beta }}_{\mathrm{full}}\right)-Var\left({\hat{\beta }}_{\mathrm{restricted}}\right)\right]}^{-1}\\ \left({\hat{\beta }}_{\mathrm{full}}-{\hat{\beta }}_{\mathrm{restricted}}\right) \end{array}$$

1. Under the null hypothesis that the IIA assumption holds, the test statistic H follows a chi-squared distribution with degrees of freedom (*df*) equal to the number of coefficients estimated $\hat{\beta }$ . If the test statistic is significantly large, the null hypothesis is rejected, indicating that the IIA assumption may not hold.

## 4 Results and discussion

### 4.1 Descriptive statistics

According to Table 3, there are slightly more females than males who joined in survey. The majority of respondents are aged from 51 to 70, married and healthy and have no problem with daily activities independently. The retired group dominates the sampled population, accounting for 81.1% of the respondents. 53.9% respondents are in the income range of 500–1,000 US dollars. Many respondents live with their partners. Close to 90% respondents have lived in this community for more than 15 years, indicating a high level of familiarity and stability with the community. Most of respondents mainly take buses and subways for daily travel as the public transportation plays an important role in urban mobility. In terms of outdoor activity duration, the residents who spend more than 60 min per day accounts for 69.7% of respondents, indicating that they have certain time arrangements for outdoor activities and pay attention to maintaining a positive lifestyle.

**Table 3:** The descriptive statistics of respondents’ personal basic information.

Variable	Class	Frequency	Percentage (%)
Gender	Male	294	46.4
	Female	340	53.6
Age	≤50	86	13.6
	51–60	142	22.4
	61–70	233	36.7
	71–80	107	16.9
	≥81	66	10.4
Marital status	Divorce	52	8.2
	Widow	101	15.9
	Unmarried	31	4.9
	Married	450	71
Health condition	Minor issues	38	6
	Commonly	107	16.9
	Good	489	77.1
Physical mobility	Difference	3	0.5
	Commonly	81	12.8
	Good	550	86.7
Work status	On the job	105	16.6
	Doing business	9	1.4
	Retire	514	81.1
	Other	6	0.9
Monthly income (USD)	≤500	276	43.5
	500–1,000	342	53.9
	1,000–1,500	14	2.2
	1,500–2000	1	0.2
	>2000	1	0.2
Composition of cohabitants	Living alone	160	25.2
	Partner	321	50.6
	Partner and Parents	5	0.8
	Partners and children	50	7.9
	Children	46	7.3
	Parent	31	4.9
	Other	21	3.3
Residential time in this community (year)	<1	3	0.5
	1–5	23	3.6
	5–10	18	2.9
	10–15	23	3.6
	>15	567	89.4
Daily travel methods	Bicycle	4	0.6
	Motorcycle	59	9.3
	Public Transportation/subway	495	78.1
	Taxi/ride hailing service	2	0.3
	Private car	74	11.7	Variable	Class	Frequency	Percentage (%)
Outdoor activity time (min/day)	≤15	11	1.8
	15–30	47	7.4
	30–45	68	10.7
	45–60	66	10.4
	>60	442	69.7

Table 4 shows the housing information of the respondents. The results of subjective evaluation of respondents on the improvements after micro-renewal of old Danwei communities are shown in Table 5. The results of SWEMWBS are shown in Table 6.

**Table 4:** The housing information of respondents.

Variable	Class	Frequency	Percentage (%)
Housing area (m^2^)	19–30	98	15.5
	30–50	359	56.5
	50–70	126	20.0
	70–90	30	4.7
	90–130	21	3.3
Housing floors	1	185	29.2
	2	172	27.1
	3	154	24.3
	4	101	15.9
	5	22	3.5
Number of bedrooms	1	87	13.7
	2	521	82.2
	3	26	4.1
Number of bathrooms	1	632	99.7
	2	2	0.3
Number of balconies	1	26	4.1
	2	608	95.9
Tenure	Own tenured	599	94.5
	Lease	28	4.4
	Other	7	1.1
Housing type	Bungalow	2	0.3
	Multi story unit room	632	99.7

**Table 5:** The evaluation on the improvements of old Danwei communities after micro-renewal.

Variable	Class	Frequency	Percentage (%)
Acoustic environment	Strongly disagree	7	1.1
	Disagree	15	2.4
	Slightly disagree	206	32.5
	Neutral	279	44
	Slightly agree	100	15.8
	Agree	27	4.3
	Strongly agree	7	1.1
Thermal environment	Strongly disagree	4	0.6
	Disagree	13	2.1
	Slightly disagree	268	42.3
	Neutral	207	32.6
	Slightly agree	116	18.3
	Agree	26	4.1
	Strongly agree	4	0.6
Air quality	Strongly disagree	1	0.2
	Disagree	10	1.6
	Slightly disagree	91	14.4
	Neutral	254	40.1
	Slightly agree	227	35.8
	Agree	51	8
	Strongly agree	1	0.2
Nighttime lighting	Strongly disagree	5	0.8
	Disagree	10	1.6
	Slightly disagree	49	7.7
	Neutral	159	25.1
	Slightly agree	306	48.3
	Agree	105	16.6
	Strongly agree	5	0.8
Grocery shopping	Strongly disagree	1	0.2
	Disagree	13	2.1
	Slightly disagree	33	5.2
	Neutral	188	29.7
	Slightly agree	184	29
	Agree	178	28.1
	Strongly agree	37	5.7

**Table 6:** The results of respondents’ mental health assessments.

Dependent variable	Frequency	Percentage (%)
Low level of mental health	95	15
Average level of mental health	243	38.3
High level of mental health	296	46.7

### 4.2 Results of entropy-TOPSIS

The weights of various indicators of satisfaction on micro-renewal of old Danwei communities are shown in Table 7. And the weights of various indicators of evaluation on improvements through micro-renewal of old Danwei communities are listed in Table 8.

**Table 7:** Weights of satisfaction regarding various micro-renewal components.

Target layer	Criterion layer	Indicator layer	Entropy	Weight	Sort
Satisfaction	Night lighting 14.08%	Corridor lighting	0.9852	6.67%	5
		Street lighting	0.9835	7.41%	2
	Facilities 19.23%	Staircase railing	0.9863	6.15%	8
		Roof waterproofing and drainage	0.9859	6.35%	6
		Road drainage and sewage	0.9850	6.73%	3
	Security 16.08%	Unit gate and access control	0.9879	5.45%	12
		Monitoring security	0.9863	6.17%	7
		Firefighting equipment	0.9901	4.46%	14
	Appearance 19.88%	Exterior wall painting	0.9873	5.71%	11
		Corridor wall painting	0.9867	6%	9
		Road pavement	0.9818	8.17%	1
	Service 12.57%	Road system	0.9850	6.73%	4
		Garbage collection	0.9870	5.84%	10
	Public space 18.16%	Fitness venues and facilities	0.9902	4.4%	16
		Public activity space	0.9903	4.37%	17
		Parking lots and facilities	0.9890	4.95%	13
		Greenery	0.9901	4.44%	15

**Table 8:** Weights of indicators for improvements in micro-renewal of old Danwei communities.

Target layer	Indicator layer	Entropy	Weight	Sort
Improvement	Acoustic environment	0.9972	10.16	6
	Thermal environment	0.9971	10.66	5
	Air quality	0.9978	8.08	8
	Nighttime lighting	0.9977	8.50	7
	Grocery shopping	0.9959	15.11	2
	Services	0.9932	24.92	1
	Public spaces	0.9968	11.90	3
	Safety status	0.9971	10.68	4

The results of comparative analysis regarding the pre and post evaluation of two old Danwei communities is shown in Table 9, where C1B and C2B denote the evaluation before micro-renewal in C1 and C2, respectively; C1A and C2A denote the condition after micro-renewal in C1 and C2, respectively; IR means the increase rate. Whether before or after micro-renewal, the overall satisfaction of respondents in C2 is higher than respondents in C1. Moreover, the average values of satisfaction on all indicators in C2 are higher than those in C1. The micro-renewal in both communities have achieved significant improvements in respondents’ satisfaction on street lighting and road pavement. In addition, more satisfaction on the improvement of monitoring and security has been found in the residents of C1. However, the improvement of gate and access control is not significant in micro-renewal of both communities. Although the overall level of satisfaction of respondents from C1 is lower than those from C2, respondents from C1 has a higher agreement with the improvement rate in many aspects than the respondents from C2. A higher improvement rate in the corridor lighting, gate and access control, and road pavement are estimated by respondents from C2 than C1. The result indicates that the micro-renewal measures in C1 is more effective in terms of residents’ satisfaction.

**Table 9:** Comparison of satisfaction before and after micro-renewal in two old Danwei communities.

Indicator layer	C1B	C1A	IR (%)	C2B	C2A	IR (%)
Corridor lighting	2.55	3.59	40.69	3.50	5.17	47.82
Street lighting	2.46	5.71	132.20	2.73	5.87	114.80
Staircase railing	2.66	4.30	61.78	3.05	4.71	54.64
Roof waterproofing and drainage	2.51	4.34	73.04	3.06	4.86	58.85
Road drainage and sewage	2.41	4.36	81.26	2.92	5.19	78.01
Exterior wall painting	2.72	4.76	75.07	2.90	4.86	67.63
Gate and access control	2.98	3.52	18.09	3.12	4.05	29.58
Firefighting equipment	2.83	4.58	61.73	3.32	4.82	45.29
Monitoring security	2.44	4.97	103.58	3.02	5.12	69.44
Corridor wall painting	2.65	4.97	87.40	2.68	4.35	62.39
Road system	2.52	4.81	91.00	2.64	4.75	79.77
Garbage collection	2.62	5.14	96.13	3.24	5.44	67.53
Road pavement	2.37	5.81	145.30	2.41	6.08	152.22
Greenery	3.14	4.99	59.02	3.80	5.09	33.94
Public activity space	3.18	5.09	59.78	3.25	5.08	56.10
Fitness venues and facilities	3.13	5.13	63.87	2.92	4.71	61.52
Parking lots and facilities	2.84	4.91	72.66	3.08	5.04	63.37
Overall satisfaction (Standardized)	0.35	0.58	67.00	0.40	0.64	56.92

**Figure 3:**
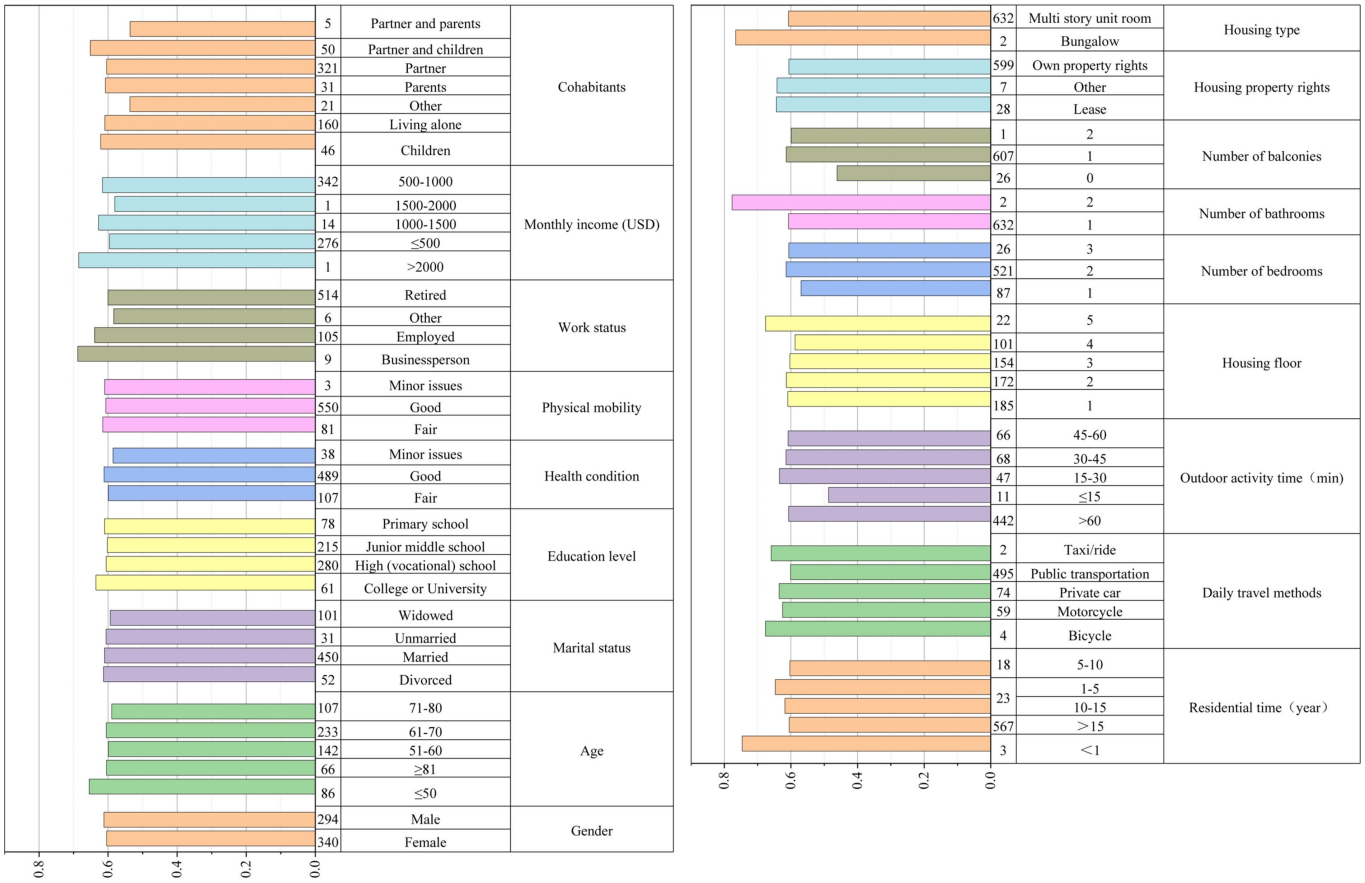
The heterogeneity of mean overall satisfaction on the micro-renewal.

As shown in Table 10, mean satisfaction level of residents on all indicators is higher in C2 than C1. The heterogeneity of satisfaction on micro-renewal is depicted in Figure 3. The results indicate that the evaluations of respondents’ satisfaction are heterogeneous in terms of eight characteristics, including of age, education level, employment, health condition, income, travel mode, apartment condition and tenure. Different age groups have varied levels of satisfaction with the micro-renewal. Older residents may place higher importance on improvements in safety and accessibility, while younger residents might prioritize enhancements in public spaces and recreational facilities. Satisfaction levels differ across education levels as well.

**Table 10:** The mean satisfaction on improvements of micro-renewal in two old Danwei communities.

Component	C1	C2
Acoustic environment	4.83	4.89
Thermal environment	4.75	4.92
Air quality	5.34	5.35
Night lighting	5.67	5.71
Grocery shopping	4.90	5.03
Services	4.14	4.57
Public space	5.20	5.21
Safety status	5.48	5.50
Overall satisfaction (Standardized)	0.60	0.63

### 4.3 Results of multinominal logit model

The goodness of fit index of MNL is estimated. The McFadden Pseudo R-squared is 0.374 that indicates a high degree of fit of the model. The H0 hypothesis, in the context of the Hausman test for the MNL model, refers to the IIA assumption, which means the choice between any two categories should be independent of the other available choices. This property is crucial for the validity of the MNL model. In the IIA hypothesis test, the result of Hausman test (see Table 11) indicates that removing one option does not affect the consistent estimation of other options, i.e., does not affect the probability of other choices. This means that MNL does not reject the IIA hypothesis. Fitting the data of this study with MNL is reasonable.

**Table 11:** Hausman test results of the MNL.

Omitted	Chi-square	*df*	*p* > chi-square	Evidence
0	−0.000	6	1.000	for H0
1	−0.000	6	1.000	for H0
2	−0.002	5	1.000	for H0

H0 hypothesis: Odds (Outcome-J vs Outcome-K) are independent of other alternatives.

As shown in Table 12, the predicted values of 465 samples are equal to the actual values, and the model has an accurate prediction rate of 73.34%, which indicates high accuracy of this model in fitting the samples.

**Table 12:** Prediction accuracy of the MNL.

Count	Frequency	Percentage (%)
−2	13	2.05
−1	87	13.72
0	465	73.34
1	63	9.94
2	6	0.95

The estimation results of MNL are shown in Table 13. Respondents with a monthly income greater than 6,000 are more likely to have high level of mental health. The probability of respondents with average mental health is relatively low when they dissatisfy on corridor wall painting of micro-renewal. The respondents are very satisfied with road pavement, and very satisfied with parking lots and facilities. Among them, the probability of a group with a monthly income greater than 6,000 having a high level of positive mental health is 4.9 times higher than that of a group with a monthly income less than 3,000. The probability of positive psychological health levels being 89.6, 3.5, and 10.1 times higher for groups who are very dissatisfied, dissatisfied, or satisfied with the unit gate and access control compared to neutral groups, respectively. The probability of being satisfied, satisfied, and very satisfied with road pavement may be 11.7, 8.7, and 14.5 times higher than that of the neutral group in terms of positive mental health. The group that is very satisfied with the renovation of parking lots and facilities may have a 10.9 times higher probability of positive mental health than the neutral group.

**Table 13:** Results of the MNL.

	Variables	Coefficient	
		Low	High
Age (Control group: Age ≤ 50)	51–60	0.621	0.067
	61–70	0.657	−0.027
	71–80	0.678	−0.018
	≥81	1.307*	−1.178*
Education level (Edu) (Control group: Edu primary school)	Junior school	−0.084	0.443
	High school or vocational school	−0.886*	0.886*
	College or university	0.369	0.685
Health condition	Good	−0.484	0.710*
Physical mobility	Good	−0.452	−0.803
Monthly income (MI) (Control group: MI < 500 USD)	500–1,000 USD	−0.109	0.188
	>1,000 USD	0.345	1.595**
Composition of cohabitants (Control group: living alone)	Partner	−0.687*	0.319
	Big family	−0.272	0.509
Outdoor activity time (OAT) (Control group: OAT<15 min/day)	15–30 min/day	−1.225	−0.913
	30–45 min/day	−1.564	−0.281
	45–60 min/day	−1.295	0.415
	>60 min/day	−2.214*	0.123
Exterior wall painting (Control group: neutral)	Very dissatisfied	−1.037	12.605
	Dissatisfied	−0.898	−2.855***
	Relatively dissatisfied	0.73	−0.57
	Relatively satisfied	0.868*	−0.257
	Satisfied	0.212	−1.120**
	Very satisfied	0.115	0.011
Corridor wall painting (Control group: neutral)	Very dissatisfied	−0.969	12.789
	Dissatisfied	−0.901	0.175
	Relatively dissatisfied	0.095	0.166
	Relatively satisfied	−1.344***	−1.196***
	Satisfied	−0.417	−0.837*
	Very satisfied	0.025	−0.268
Corridor lighting (Control group: neutral)	Very dissatisfied	−0.276	−0.007
	Dissatisfied	−1.344**	0.869*
	Relatively dissatisfied	−1.377*	0.261
	Relatively satisfied	−0.004	0.634*
	Satisfied	0.071	0.701
	Very satisfied	−14.58	1.272
Gate and access control (Control group: neutral)	Very dissatisfied	1.08	4.496***
	Dissatisfied	−0.17	1.276**
	Relatively dissatisfied	0.058	0.069
	Relatively satisfied	−0.633	−0.041
	Satisfied	0.134	2.316**
	Very satisfied	0.739	1.366
Road pavement (Control group: neutral)	Very dissatisfied	19.502	4.879
	Dissatisfied	1.09	2.096
	Relatively dissatisfied	0.575	0.426
	Relatively satisfied	0.392	2.461***
	Satisfied	−0.393	2.169***
	Very satisfied	−0.711	2.677***
Road system (Control group: neutral)	Very dissatisfied	−13.191	14.804
	Dissatisfied	1.344	−1.503*
	Relatively dissatisfied	1.678**	−0.204
	Relatively satisfied	0.657	−1.026***
	Satisfied	0.64	−2.266***
	Very satisfied	1.328	−1.537**
Public activity space (Control group: neutral)	Dissatisfied	0.592	0.846
	Relatively dissatisfied	−1.723*	−0.524
	Relatively satisfied	−0.222	−0.321
	Satisfied	−0.904	−0.345
	Very satisfied	−0.763	−2.318***
Fitness venues and facilities (Control group: neutral)	Dissatisfied	16.653	14.524
	Relatively dissatisfied	−0.49	0.621
	Relatively satisfied	−0.361	−0.313
	Satisfied	0.226	0.072
	Very satisfied	−1.132	−0.314
Parking lots and facilities (Control group: neutral)	Dissatisfied	−0.979	−18.799
	Relatively dissatisfied	−1.006*	−1.837***
	Relatively satisfied	−0.436	0.238
	Satisfied	−0.523	0.17
	Very satisfied	1.205	2.396***
	Constant	2.508	−1.394

***p < 0.01, **p < 0.05, *p < 0.1.

As shown in Table 14, the probability of being low level of mental health increase by 16.6% if the respondents are over 80-year-old. Aging significantly decreases the mental health of residents living in the old Danwei community. The relatively higher education level leads to higher probability of respondents to be high level of mental health. The respondents with good physical health are more likely to being high level mental health. The respondents having greater than 6,000-yuan monthly income are 19.5% increase in the possibility of being high mental health level.

**Table 14:** The marginal effects in MNL.

Variable		Low	Average	High
Age (classification) (Control group: ≤ 50)	51–60	0.045	−0.036	−0.009
	61–70	0.051	−0.028	−0.023
	71–80	0.053	−0.03	−0.022
	≥81	0.166**	0.025	−0.191**
Education level (Control group: primary school)	Junior high school	−0.025	−0.034	0.059
	High school, vocational school	−0.107**	−0.037	0.143**
	College or university	0.015	−0.087	0.072
Health condition	Good	−0.066*	−0.042	0.109**
Physical mobility	Good	−0.014	0.105	−0.09
Monthly income (Control group:<3000)	3,000–6,000	−0.016	−0.012	0.028
	>6,000	−0.029	−0.166*	0.195**
Composition of cohabitants (Control group: living alone)	Partner	−0.076**	0.011	0.065
	Big family	−0.047	−0.03	0.077
Outdoor activity time (Control group:<15)	15–30	−0.125	0.171	−0.045
	30–45	−0.191	0.146	0.045
	45–60	−0.19	0.065	0.125
	>60	−0.264*	0.145	0.119
Exterior wall painting (Control group: neutral)	Very dissatisfied	−0.104	−0.328	0.432
	Dissatisfied	−0.008	0.332***	−0.324***
	Relatively dissatisfied	0.081	0.02	−0.102
	Relatively satisfied	0.083**	−0.018	−0.065
	Satisfied	0.048	0.108*	−0.157**
	Very satisfied	0.008	−0.006	−0.002
Corridor wall painting (Control group: neutral)	Very dissatisfied	−0.174	−0.275	0.449
	Dissatisfied	−0.079	0.023	0.056
	Relatively dissatisfied	0.002	−0.02	0.018
	Relatively satisfied	−0.075*	0.198***	−0.122**
	Satisfied	−0.008	0.107*	−0.098
	Very satisfied	0.014	0.024	−0.038
Corridor lighting (Control group: neutral)	Very dissatisfied	−0.028	0.019	0.009
	Dissatisfied	−0.125***	−0.03	0.155**
	Relatively dissatisfied	−0.116***	0.043	0.072
	Relatively satisfied	−0.025	−0.06	0.085*
	Satisfied	−0.021	−0.071	0.091*
	Very satisfied	−0.191	−0.041	0.232
Unit gate and access control (Control group: neutral)	Very dissatisfied	−0.101**	−0.364***	0.465***
	Dissatisfied	−0.059	−0.126*	0.185***
	Relatively dissatisfied	0.003	−0.011	0.007
	Relatively satisfied	−0.055	0.041	0.013
	Satisfied	−0.074	−0.233**	0.308***
	Very satisfied	0.015	−0.175	0.159
Road pavement (Control group: neutral)	Very dissatisfied	0.726	−0.546	−0.18
	Dissatisfied	0.068	−0.249	0.181
	Relatively dissatisfied	0.067	−0.088	0.021
	Relatively satisfied	−0.038	−0.225**	0.263***
	Satisfied	−0.105	−0.149*	0.254***
	Very satisfied	−0.144*	−0.187**	0.330***
Road planning for pedestrian and vehicular traffic diversion (Control group: neutral)	Very dissatisfied	−0.080***	−0.265	0.345
	Dissatisfied	0.178	0.092	−0.270*
	Relatively dissatisfied	0.149*	−0.048	−0.101
	Relatively satisfied	0.082*	0.091	−0.173***
	Satisfied	0.123**	0.219***	−0.341***
	Very satisfied	0.178	0.096	−0.274**
Public activity space (Control group: neutral)	Dissatisfied	0.021	−0.103	0.081
	Relatively dissatisfied	−0.116	0.138	−0.023
	Relatively satisfied	−0.01	0.043	−0.033
	Satisfied	−0.069	0.084	−0.016
	Very satisfied	−0.009	0.258*	−0.249**
Fitness venues and facilities (Control group: neutral)	Dissatisfied	0.401**	−0.377***	−0.024
	Relatively dissatisfied	−0.058	−0.04	0.098
	Relatively satisfied	−0.022	0.052	−0.03
	Satisfied	0.019	−0.02	0.001
	Very satisfied	−0.076	0.089	−0.012
Parking lots and facilities (Control group: neutral)	Dissatisfied	0	0.435	−0.436
	Relatively dissatisfied	−0.048	0.240***	−0.192***
	Relatively satisfied	−0.047	0.001	0.046
	Satisfied	−0.052	0.013	0.04
	Very satisfied	−0.001	−0.252***	0.253**

***p ≤ 0.01, **0.01 *< p* ≤ 0.05, *0.05 < *p* ≤ 0.1.

The possibility to be lower level of mental health is increased by 8.3% if respondents who are more satisfied with exterior wall painting. The probability of high-level mental health increases by 15.5% if respondents dissatisfy with corridor lighting. The probability to have high level of mental health increased by 30.8% if respondents satisfy with the gate and access control after micro-renewal. The satisfaction on road pavement and road system will also increase the probability of high-level mental health. However, the dissatisfaction with fitness venues and facilities results in more likely to be lower level of mental health increased. The probability of being high level of mental health increases by 25.3% if respondents are satisfied with the parking lot and facilities.

The sensitivity analysis was performed that highlights the importance of demographic and health-related factors in predicting mental health outcomes. Males have a slightly higher probability of low mental health compared to females. As age increases, the probability of low mental health increases. Individuals who are divorced have higher probability of high mental health against those who are married, unmarried, or widowed. Moreover, respondents with higher education levels, better health status are associated with higher probabilities of high mental health in old Danwei community. It suggests that interventions targeting these factors could potentially improve mental health of residents in old Danwei communities.

### 4.4 Discussion

The results of this study provide important insights into the factors influencing residents’ satisfaction and mental health in the context of micro-renewal in old Danwei communities. The Entropy-TOPSIS analysis highlighted that improvements in services, road systems, and grocery shopping were the most critical components contributing to overall satisfaction with the micro-renewal. The highest weights were assigned to these components indicate that functional infrastructure and basic services are fundamental to enhancing the quality of life in aging communities. These findings align with existing literature on urban renewal, which emphasizes that physical improvements in infrastructure are crucial for resident satisfaction and well-being (13, 30).

The emphasis on services and infrastructure improvements, particularly in areas like garbage collection and road systems, suggests that residents prioritize practical, everyday necessities. These components not only address basic needs but also enhance the overall livability of the community, which is critical for fostering a sense of security and stability among residents. Public spaces and safety measures were also significant, reflecting the importance of community interactions and a safe living environment in supporting mental health. This resonates with theories of environmental psychology, which posit that well-maintained, safe environments contribute to reduced stress and improved mental well-being (37).

The findings from MNL model indicate that socio-demographic factors such as age, income, and health condition play a significant role in determining residents’ mental health outcomes. Older residents and those with higher incomes were more likely to report better mental health, suggesting that financial stability and life experience may provide a buffer against the stressors associated with aging in a deteriorating environment. This finding supports the life course perspective, which suggests that individuals’ health trajectories are shaped by a combination of socio-economic factors and environmental exposures over time (38).

The differentiated impacts of micro-renewal on various demographic groups underscore the need for tailored interventions in old Danwei community renewal. The study suggests that while infrastructural improvements benefit all residents, specific demographic groups may have unique needs that require targeted approaches. For example, older residents may benefit more from improvements in accessibility and safety, while younger populations might prioritize recreational facilities and modern conveniences. This aligns with the concept of “proportionate universalism,” which advocates for universal interventions that are scaled according to the level of disadvantage (39).

The findings suggest that urban renewal strategies in old Danwei communities should prioritize inclusive planning processes that actively engage diverse community members. By involving residents in the planning and decision-making processes, micro-renewal projects can better address the specific needs and preferences of different demographic groups, ultimately leading to higher satisfaction and better mental health outcomes. This participatory approach is particularly important in the context of micro-renewal, where the scale of intervention allows for more community-driven decision-making (40).

The findings from both models highlight the critical role of infrastructure improvements in enhancing residents’ satisfaction and mental health. Specifically, the high weights and significant variables related to road system, lighting, and public safety suggest that these elements are vital for improving the quality of life in the old Danwei communities. The influence of socio-demographic factors on mental health outcomes underscores the need for tailored approaches in old Danwei community renewal. Different demographic groups have varying needs and responses to environmental changes, as evidenced by the differentiated impacts observed in this study. It is highly recommended that adopting inclusive planning processes and engaging diverse community members when it comes to the old community renewal. Tailoring intervention to meet the specific needs of different demographic groups, such as providing accessible public spaces for older residents and enhancing recreational facilities for younger population, can maximize the benefits of community renewal.

The findings suggest that urban renewal strategies in old Danwei communities should prioritize inclusive planning processes that actively engage diverse community members. By involving residents in the planning and decision-making processes, micro-renewal projects can better address the specific needs and preferences of different demographic groups, ultimately leading to higher satisfaction and better mental health outcomes. This participatory approach is particularly important in the context of micro-renewal, where the scale of intervention allows for more community-driven decision-making.

## 5 Conclusion

This study examines the impact of micro-renewal on the mental health of residents in old Danwei communities in Hengyang, China. The findings indicate that socio-demographic characteristics and behavior factors significantly influence the mental health of residents. The satisfaction with various components of micro-renewal, such as road pavement, unit gate and access control, and parking facilities, plays a crucial role in determining the mental health levels of residents. High satisfaction levels in these areas are associated with better mental health outcomes.

The research highlights the importance of tailored approaches in community renewal to meet the specific needs of different demographic groups. Engaging diverse community members and adopting inclusive planning processes are essential for maximizing the benefits of neighborhood renewal. The study also emphasizes the need for longitudinal empirical research to better understand the long-term impact of micro-renewal on mental health.

Several limitations primarily revolve around data collection, modeling assumptions, and generalizability need to be addressed. Although there is a key assumption that the results from Hengyang could be broadly applicable to other Danwei communities in different cities in China. This assumption may not hold if other regions have significantly different socio-economic dynamics, urban policies, or cultural contexts. Hence, our future study will include multiple cities with diverse characteristics to enhance the generalizability of the findings. The cross-sectional data used in this study captures no information regarding the long-term impact of micro-renewal on mental health and changes over time.

## Data Availability

The raw data supporting the conclusions of this article will be made available by the authors, without undue reservation.
